# *Akkermansia muciniphila* supplementation improves glucose tolerance in intestinal *Ffar4* knockout mice during the daily light to dark transition

**DOI:** 10.1128/msystems.00573-23

**Published:** 2023-10-03

**Authors:** Zhe Wang, Siyuan Cui, TingTing Zhang, Wei Wang, JiaYu Li, Y. Q. Chen, Sheng long Zhu

**Affiliations:** 1 Jiangnan University Medical Center, Wuxi School of Medicine, Jiangnan University, Wuxi, China; 2 School of Food Science and Technology, Jiangnan University, Wuxi, China; University of California San Diego, La Jolla, California, USA

**Keywords:** FFAR4, glucose tolerance, *Akkermansia muciniphila*, GLP-1

## Abstract

Disruption of blood glucose utilization may lead to diabetes mellitus, which has complex genetic and environmental aspects, and free fatty acid receptors (FFARs) may bridge the genetic and dietary aspects. FFAR4 has been identified as a new target for diabetes treatment, and it is essential to investigate how FFAR4 affects glucose homeostasis. FFAR4 knockout mice have been shown to cause severely impaired glucose tolerance under high-fat feeding conditions; however, the findings in FFAR4 knockout mice under chow diet conditions seem to be contradictory. Blood glucose utilization in mice under chow diet conditions is thought to show diurnal rhythmicity. In this study, we aim to investigate the role of FFAR4 in glucose utilization rhythm and explore the possible mechanism. Total *Ffar4* and gut-specific *Ffar4* knockout mice both showed a clear diurnal rhythm in glucose tolerance. However, deletion of total *Ffar4* and gut-specific *Ffar4* both deteriorate glucose tolerance at the daily light to dark transition (ZT12) in mice. We show that intestinal FFAR4 deficiency leads to significant changes in fecal microbiota at different ZTs. 16S rRNA sequencing results suggested that *Akkermansia muciniphila* was the main target of intestinal FFAR4 at ZT12. *Akkermansia muciniphila* supplementation significantly improved the impaired glucose tolerance at ZT12 in mice with gut-specific *Ffar4* deletion and was accompanied by recovery of serum hormone level and transcripts of key genes in glucose metabolism.

**IMPORTANCE:**

Alterations in the intestinal environment are associated with various diseases, and FFAR4 is abundantly enriched in the intestine, where it has been shown to have the ability to regulate intestinal hormone secretion and intestinal microbiota; here, we confirmed previous reports. Meanwhile, we found that intestinal FFAR4 regulates glucagon-like peptide 1 secretion by decreasing *Akkermansia muciniphila* abundance and show that such change is associated with the level of glucose utilization at ZT12 in mice. Intestinal FFAR4 deficiency leads to severely impaired glucose tolerance at the ZT12 moment in mice, and *Akkermansia muciniphila* supplementation ameliorates the abnormal glucose utilization at the ZT12 moment caused by FFAR4 deficiency, which is very similar to the dawn phenomenon in diabetic patients. Collectively, our data suggest that intestinal *Ffar4* deteriorates glucose tolerance at the daily light to dark transition by affecting *Akkermansia muciniphila*.

## INTRODUCTION

The worldwide epidemic of type 2 diabetes has become a major challenge for public health (1). The most significant concern in the prevention and treatment of type 2 diabetes is the daily management of blood glucose (2). In healthy individuals, insulin sensitivity is significantly higher at the time of awakening than during sleep, which leads to increased glucose tolerance while awakening (3 – 6). Patients with diabetes frequently experience the “dawn phenomenon” (DP), wherein blood glucose or insulin demand increases spontaneously at dawn despite their efforts to lower blood glucose levels (7 – 9). One possible mechanism is impairment in the rhythm of glucose tolerance (9, 10). Little is known about its precise mechanism and the key players governing the glucose utilization rhythm. ZT12 would be the equivalent of dawn for mice since mice are nocturnal. Therefore, the change of glucose tolerance in mice at ZT12 is an important node in the study of dawn phenomenon (9).

G protein-coupled receptors play an important role in regulating glucose metabolism and have been implicated in the development and progression of diabetes (11 – 13). Among them, the free fatty acid receptor (FFAR) FFAR1 (GPR40), FFAR2 (GPR43), FFAR3 (GPR41), and FFAR4 (GPR120) may bridge the genetic and environmental aspects of diabetes (14 – 18). FFAR2 and FFAR3 are activated by short-chain fatty acids (SCFAs) (19), whereas FFAR1 and FFAR4 are activated by medium-chain and long-chain fatty acids (LCFAs) (20, 21). Fatty acids, especially the LCFAs, are major components of the human diet.

FFAR4 is highly expressed in the intestine and primarily serves as the receptor for LCFAs (22 – 24). Studies show that LCFAs regulate the peripheral clock (25). In addition, the circulating level of FGF21, a key downstream regulator of FFAR4, also participates in the rhythm of glucose metabolism (26 – 28). The therapeutic effect of FGF21 on insulin sensitivity has been widely reported. Thus, it is reasonable to hypothesize that FFAR4 may participate in the rhythm of glucose tolerance. Therefore, the role of FFAR4, if any, in improving the rhythm of glucose metabolism deserves attention.

In this study, we found FFAR4 expression shows diurnal oscillations in mice colons, and intestinal *Ffar4* deficiency significantly reduced the level of glucose utilization at the daily light to dark transition. Moreover, we found that the impaired glucose tolerance at the daily light to dark transition induced by *Ffar4* deletion was mediated by *Akkermansia muciniphila*.

## RESULTS

### *Ffar4* deficiency impairs the glucose tolerance at certain moments

**FIG 1:**
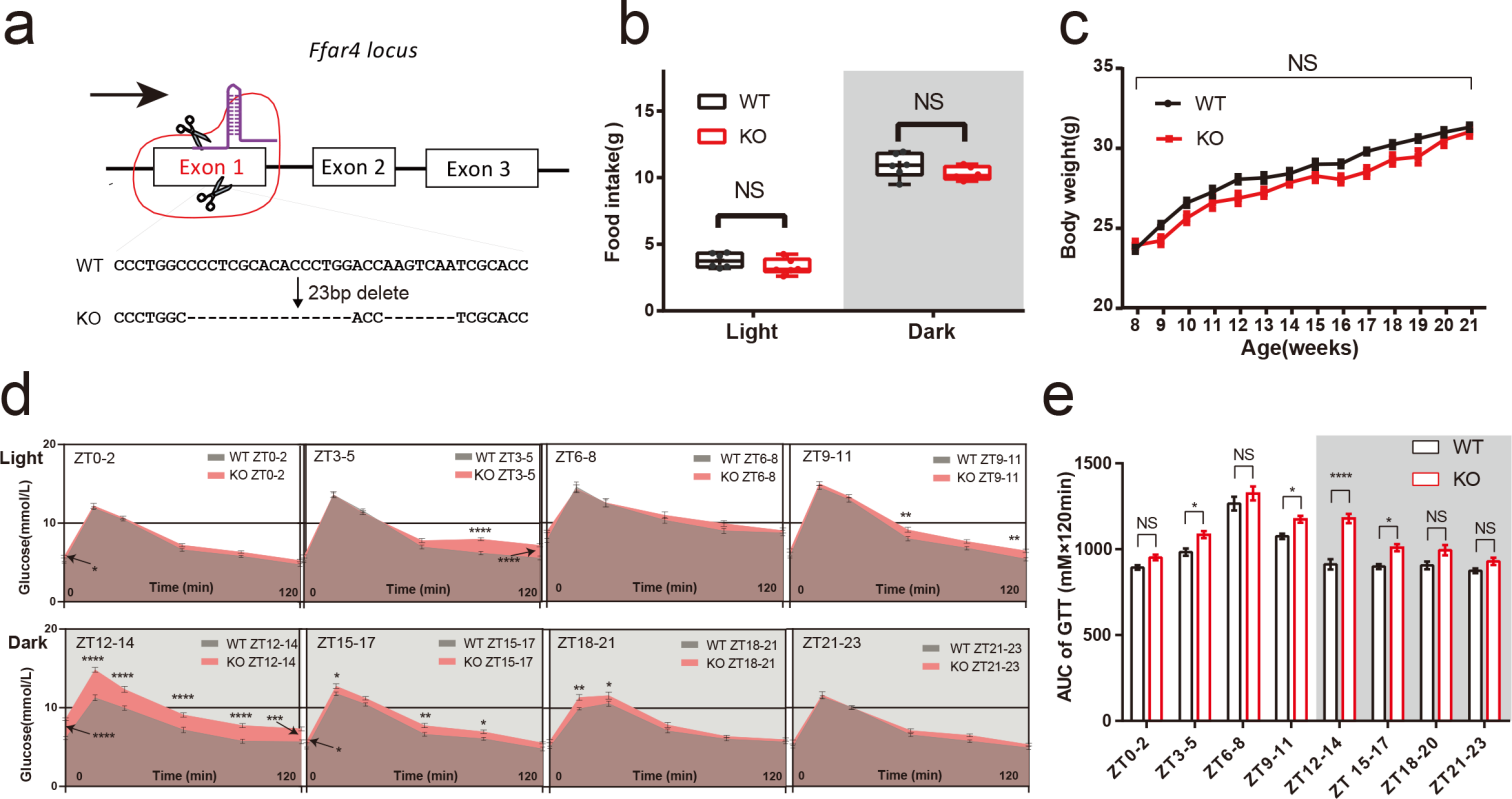
*Ffar4* knockout significantly impaired glucose tolerance at ZT12-14. CRISPR–Cas9 gene editing strategy for total *Ffar4* KO mice. Genomic sequencing identified the deletion of 16- and 7-bp nucleotides from the first exon of *Ffar4* (a). Day and night food intake (WT/KO mice, *n* = 6) (b), body weight per week (WT/KO mice, *n* = 10) (c) between WT and KO mice were recorded. Blood glucose at each time point in GTT (d), AUCs at each ZT (e) between WT and KO mice were performed using two-way ANOVA (WT/KO mice, *n* = 13 per ZT). Data are expressed as the mean ± standard error of the mean. *P* < 0.05 was considered statistically significant using two-way ANOVA. * represents *P* < 0.05；** represents *P* < 0.01；*** represents *P* < 0.001；**** represents *P* < 0.0001, ANOVA, analysis of variance; KO, knockout; NS, not significant; WT, wild type.

Glucose uptake showed a clear circadian rhythm, which is directly due to glucose-induced differences in insulin release from the islet β cells or the daily variation in tissue sensitivity to insulin (3). *FFAR4* has gained considerable recent attention due to its antidiabetic ability (29, 30). To reveal the specific role of *Ffar4* in the rhythm of glucose uptake, a mouse model of total *Ffar4* knockout (KO) was established to study whether *Ffar4* deletion affected the rhythm of glucose uptake (Fig. 1a; Fig. S1a). KO mice showed no effect on their food intake or body weight compared to wild-type (WT) mice under normal chow diet (Fig. 1b and c). On 24-hour glucose detection, there was no difference between WT and KO mice, except for a decrease in blood glucose at the ZT0 moment in the KO mice (*P* < 0.01) (Fig. S1c). Analysis of glucose tolerance test (GTT) at eight time points revealed that glucose tolerance of both WT and KO mice showed significant diurnal rhythmicity [cosine analysis was performed using areas under curves (AUC): acrophase ZT6.94, bathyphase ZT18.94 (*P*_cosinor_ < 0.05); acrophase ZT8.27, bathyphase ZT20.27 (*P*_cosinor_ < 0.001), respectively] (Fig. S1b, d, and e). Notably, KO mice showed a slight impairment of glucose tolerance at ZT3–5; ZT9–11; ZT15–17 (*P* < 0.05), a robust impairment at ZT12–14 compared with WT mice (*P* < 0.0001), which is the main cause of the delay of the acrophase and the bathyphase (Fig. 1d and e; Fig. S1d and e). Additionally, analysis of serum metabolism-related hormones revealed that KO mice did not show an altered rhythmicity in their levels of blood insulin, glucagon, and glucocorticoid (GC) but had different levels of growth hormone (GH) at certain ZTs, compared to wild-type mice (S1f-i). Notably, glucagon-like peptide 1 (GLP-1) levels decreased significantly in KO mice at ZT0, ZT6, ZT12, and ZT18 (all *P* < 0.01) (S1j).

### Intestinal loss of *Ffar4* recapitulates the attenuation of glucose tolerance at ZT12

**FIG 2:**
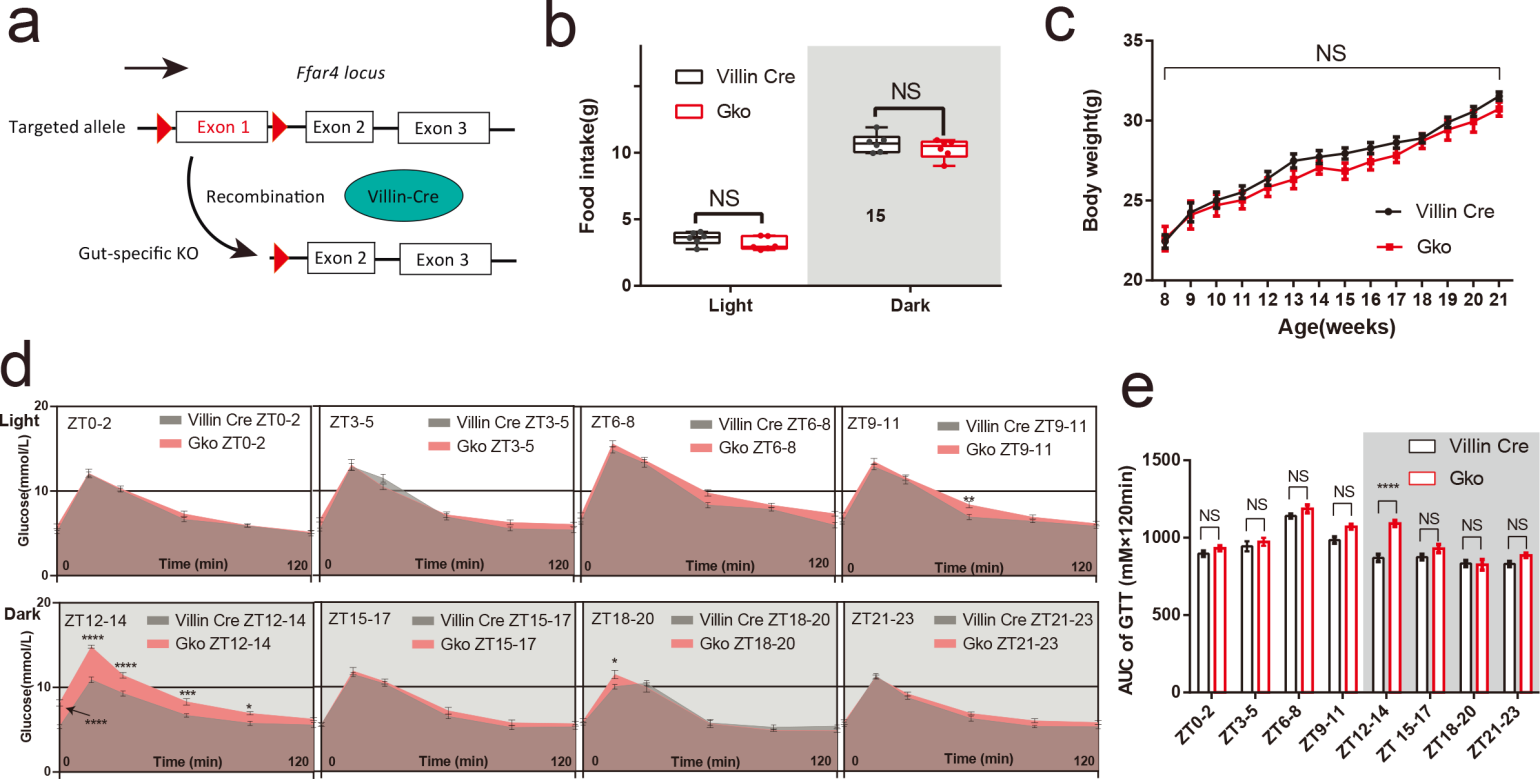
Gut-specific knockout of *Ffar4* can result in aberrant glucose tolerance at ZT12–14. Gene targeting strategy for gut-specific *Ffar4* knockout (Gko). Mice with floxed exon 1 of *Ffar4* were generated by homologous recombination. Gko mice were obtained by mating with Villin-Cre mice (a). Day and night food intake (Villin-Cre/Gko mice, *n* = 6) (b), body weight per week (Villin-Cre/Gko mice, *n* = 6) (c). Blood glucose at each time point in GTT (d), AUCs at each ZT (e) between Villin-Cre and Gko mice were performed using two-way ANOVA (Villin-Cre/Gko mice, *n* = 8 per ZT). Data are expressed as the mean ± standard error of the mean. *P* < 0.05 was considered statistically significant using two-way ANOVA; * represents *P* < 0.05；** represents *P* < 0.01；*** represents *P* < 0.001；**** represents *P* < 0.0001; NS, not significant.

To determine whether intestinal *Ffar4* is the main contributor to impairment glucose tolerance, a Gko mouse model was established (Fig. 2a; Fig. S3a). Consistent with the findings in the *Ffar4* total KO mice, there were no significant differences in daily food intake, body weight, and 24-hour glucose between Gko mice and Villin-Cre mice (Fig. 2b and c; Fig. S3d). Gko mice also exhibited impaired tolerance at ZT12–14 (Fig. 2d and e). Moreover, glucose tolerance of both Villin Cre and Gko mice showed significant diurnal rhythmicity [cosine analysis was performed using AUC: acrophase ZT6.36, bathyphase ZT18.36 (*P*_cosinor_ < 0.05); acrophase ZT7.71, bathyphase ZT19.71 (*P*_cosinor_ < 0.001), respectively] (Fig. S3b and c). Gko mice did not have significant difference levels in blood insulin, glucagon, GC, and GH at any ZTs compared to Villin Cre mice; the difference in GLP-1 levels between Villin Cre and Gko mice was still the most prominent (Fig. S3e and i). These results indicate that loss of intestinal FFAR4 is primarily responsible for the attenuation of the glucose tolerance at ZT12.

### FFAR4 deficiency causes changes in the intestinal environment of mice

**FIG 3:**
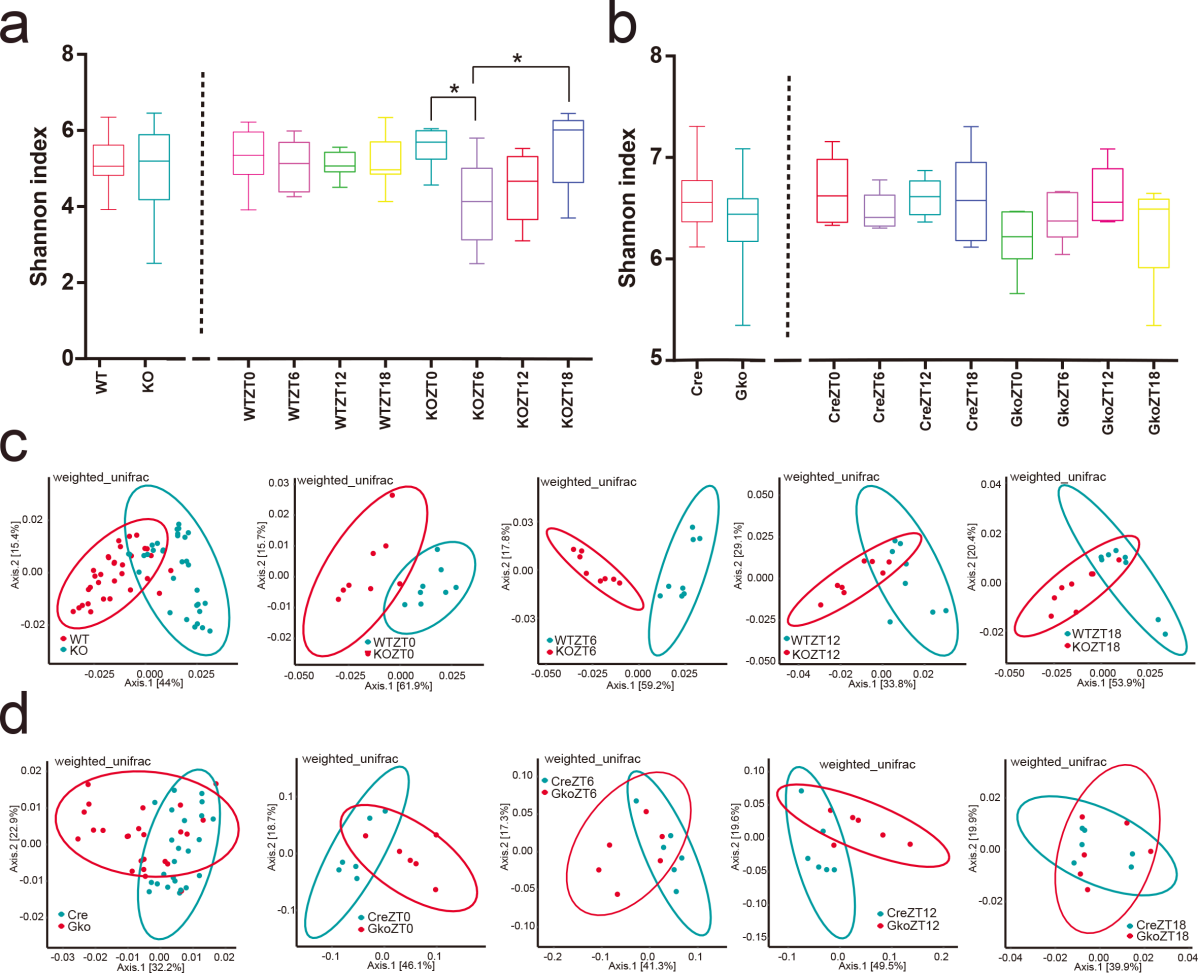
FFAR4 deletion induces changes in the alpha and beta diversity of mice intestinal microbes. The alpha diversity (shannon Index) of WT, Villin-Cre, KO, Gko mice were shown in (**a and **b), * represents *P* < 0.05. Beta diversity analysis (weighted unifrac) is shown in **panels b–**d (WT/KO mice, *n* = 8 per ZT; Villin-Cre/Gko mice, *n* = 6 per ZT).

FFAR4 is abundantly distributed in the gut and has been shown to regulate the composition of the intestinal microbiota (31, 32). A variety of gut microbes have been reported to improve glucose metabolism in mice (33, 34). To investigate why FFAR4 deficiency causes impaired glucose tolerance, we analysed the fecal fractions of four genotypes of mice at ZT0, ZT6, ZT12, and ZT18. The alpha diversity of KO mice shows a significant change at the different time points. However, 16S rRNA sequencing analysis from a combined or single ZT both showed no significant differences in microbiota alpha diversity in WT/KO mice (Fig. 3a; Fig. S4c and d) and Villin Cre/Gko mice (Fig. 3b; Fig. S4e and f). In contrast, beta diversity analysis showed that both groups of mice (WT and KO mice; Villin Cre and Gko mice) had a clear separation either individually or combined (Fig. 3c and d). In addition, beta diversity did not change over time at any mouse genotype (Fig. S4g and h). Moreover, mice fecal SCFAs showed that the levels of acetic acid in KO mice increased compared to wild-type mice at ZT12, but no significant differences in propionic and butanoic acid levels at ZT12 (Fig. S4a). The levels of acetic acid, propionic acid, and butyric acid in the feces of Gko mice were not significantly different from those of Villin Cre mice at ZT12 (Fig. S4b).

### *Akkermansia muciniphila* is a target of *Ffar4* deletion-induced aberrant glucose homeostasis

**FIG 4:**
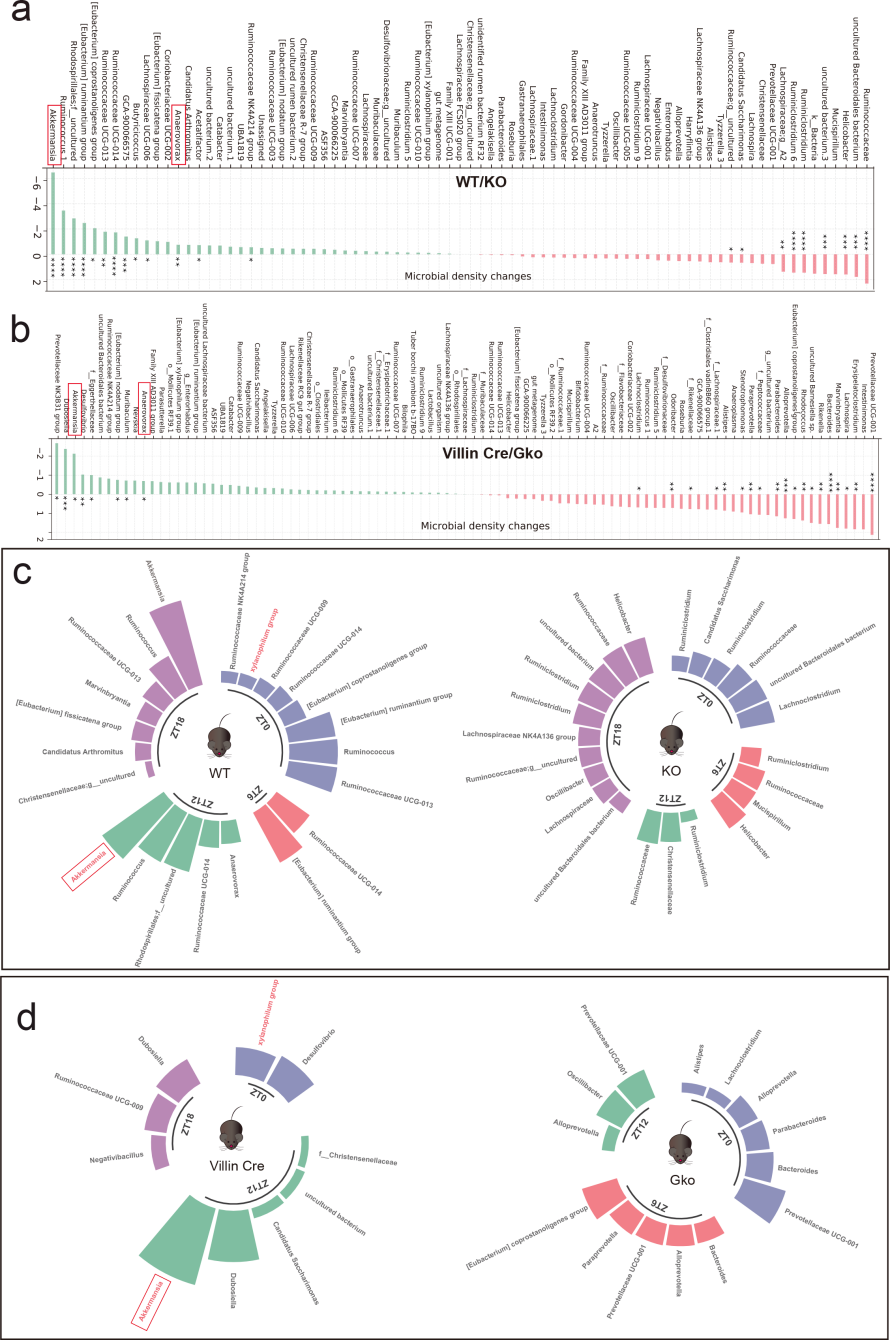
Deletion of gut *Ffar4* led to a decrease in the abundance of *Akkermansia muciniphila*. QMD was used to show the most significant dominant microbial species in the two groups of mice (**a and **b) at different ZTs (**c and **d) (WT/KO mice, *n* = 8 per ZT; Villin-Cre/Gko mice, *n* = 6 per ZT). KO, knockout; WT, wild type. * represents *P* < 0.05；** represents *P* < 0.01；*** represents *P* < 0.001；**** represents *P* < 0.0001.

Next, quantification of microbial absolute abundance differences (QMD) was used to identify the dominant microbes at different time points; *g_Akkermansia muciniphila* was found to be the most dominant groups in the intersection of two groups of mice (Fig. 4a and b). As the microbiome has circadian rhythmicity, it is worth mentioning that *g_Akkermansia muciniphila* is the most different taxa in WT and KO mice at ZT12 and ZT18 moments (Fig. 4c) and in Villin Cre and Gko mice at ZT12 (Fig. 4d). Moreover, both total *Ffar4* deletion and intestinal *Ffar4* deletion decreased the abundance levels of *Akkermansia muciniphila* (*A. muciniphila*), as detected by quantitative real-time PCR (qPCR) at ZT12 and ZT18 (Fig. S5a and b). Studies by Chiang et al. (35) and Plovier et al. (36) have demonstrated that *A. muciniphila* is sufficient to improve the metabolic syndromes, such as type 2 diabetes mellitus and obesity (35, 36). Shin et al. (37) found that the oral administration of *A. muciniphila* to mice reversed high-fat diet-induced obesity and improved glucose tolerance when fed a high-fat diet (37). These results indicate that intestinal *Ffar4* deletion significantly reduces the absolute abundance of *A. muciniphila*, which may be a key gut microbiota in maintaining the glucose homeostasis.

### *A. muciniphila* supplementation improves abnormal glucose tolerance in Gko mice

**FIG 5:**
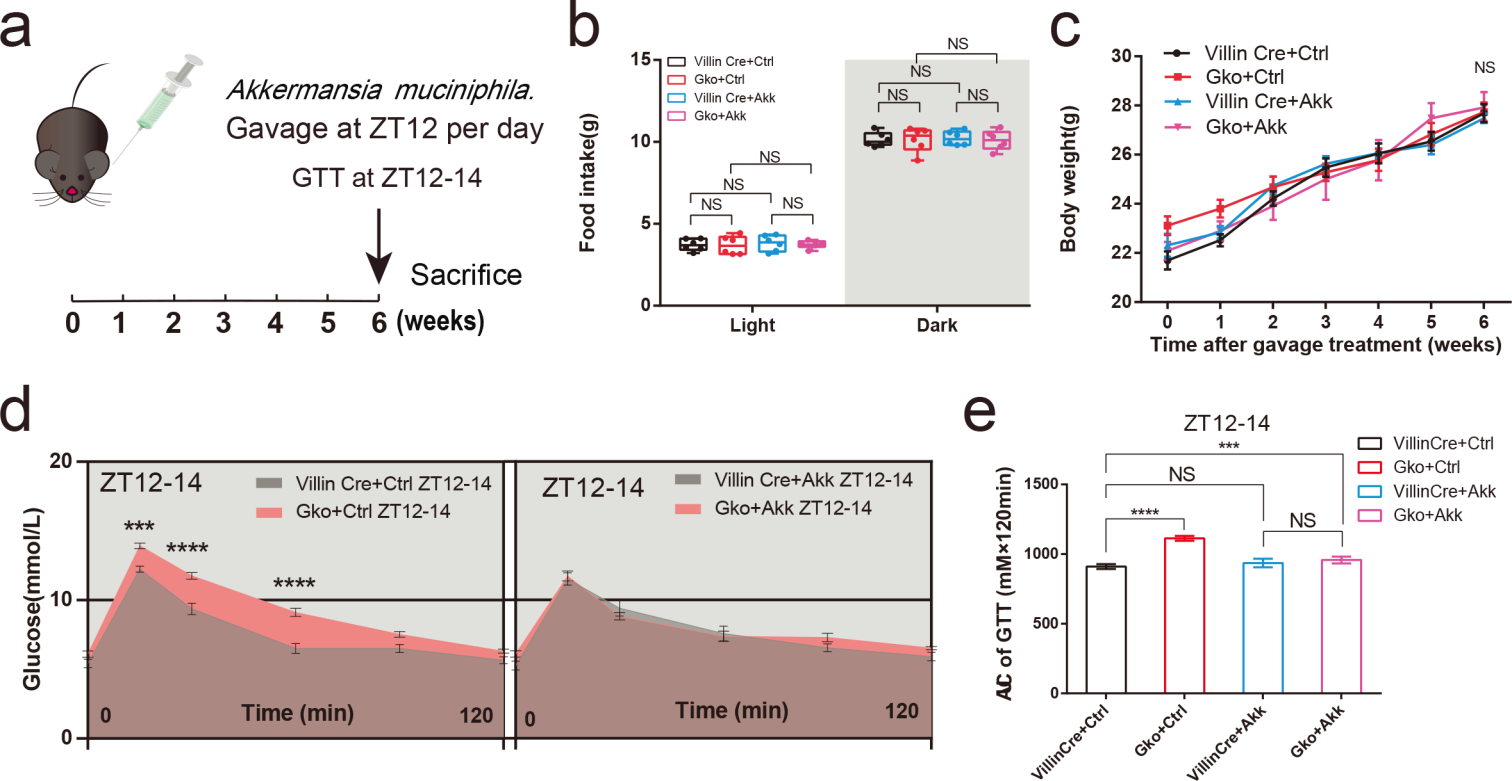
Pasteurized *A. muciniphila* can improve impaired glucose tolerance at ZT12 in gut-specific *Ffar4* knockout (Gko) mice. Gko mice were gavaged with pasteurized *A. muciniphila* (Akk) for 6 weeks, and the glucose tolerance test was conducted at ZT12–14 in the 6th week (a). Day and night food intake (b), body weight per week (c) between four groups of mice were recorded (Villin Cre + Ctrl/Villin Cre + Akk/Gko + Ctrl/Gko + Akk, *n* = 7). Blood glucose at each time point in GTT were performed using two-way ANOVA (d), AUCs at ZT12–14 (e) between WT and KO mice were performed using one-way ANOVA (Villin Cre + Ctrl/Villin Cre + Akk/Gko + Ctrl/Gko + Akk, *n* = 7) (**d and **e). Data are expressed as the mean ± standard error of the mean. Interaction effects analysis was used to analyze differences between groups (*P* < 0.05 was considered statistically significant). *** represents *P* < 0.001；**** represents *P* < 0.0001; NS, not significant.

To determine the specific role of *A. muciniphila* in aberrant glucose tolerance induced by intestinal *Ffar4* deletion, Villin Cre and Gko mice were gavaged with a pasteurized solution of *A. muciniphila* at ZT12 for 6 weeks (Fig. 5a). Within 6 weeks of treatment, there were no significant differences in body weights and the daily food intake of mice compared with those in the control group (Fig. 5b and c). After administration of the oral gavage of pasteurized *A. muciniphila* solution, Gko mice showed significant improvements in glucose tolerance at ZT12–14 but not in Villin Cre mice. The differences between Gko + Ctrl group and Gko + Akk group were considered significant (*P* < 0. 001) (Fig. 5d and e). Compared to the control group (Gko + Ctrl), treatment with *A. muciniphila* significantly increased serum insulin and GLP-1 level (Fig. S6a and e) and decreased glucagon and GH level of Gko mice (Fig. S6b and d) . Insulin levels were upregulated in Villin Cre mice at the ZT12 after pasteurized *A. muciniphila* gavage compared to the control group (Villin Cre + Ctrl) (Fig. S6a). Although the upregulation of GLP-1 after pasteurized *A. muciniphila* gavage was not significant, the significant difference in serum GLP-1 levels at ZT12 between Villin Cre and Gko mice was diminished by pasteurized *A. muciniphila* gavage (Fig. S6e).

### *A. muciniphila* supplementation improves key transcripts of glucose metabolism at ZT12

**FIG 6:**
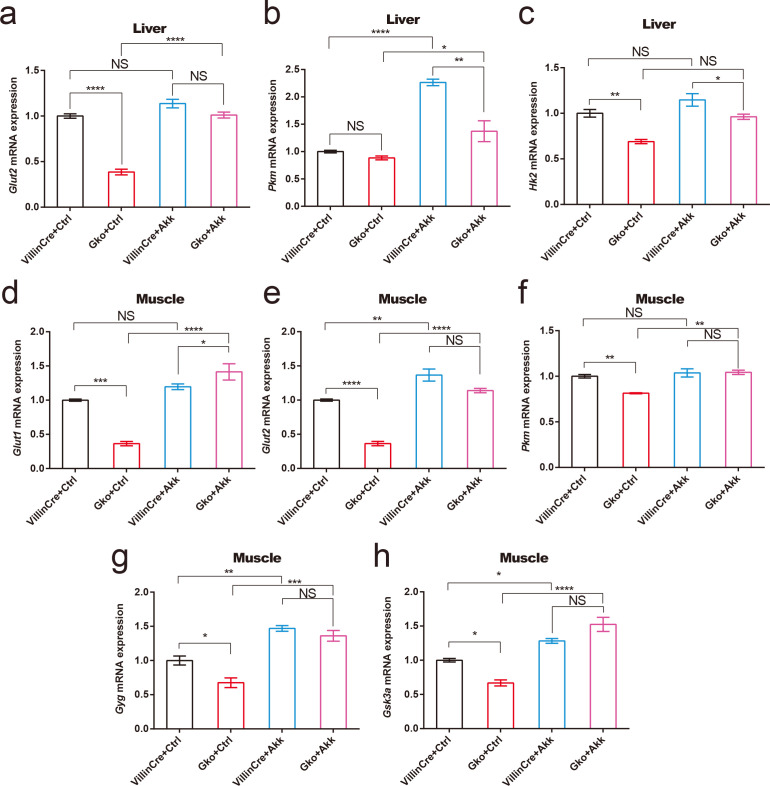
Pasteurized *A. muciniphila* can improve transcripts of key genes in glucose metabolism at ZT12 in gut-specific *Ffar4* knockout (Gko) mice. Glucose metabolism transcript changes in liver (*Glut2*, *Pkm*, and *Hk2*) and muscle (*Glut1*, *Glut2*, *Pkm*, *Gyg*, and *Gsk3a*) are shown in panels **a–**h. Data are expressed as the mean ± standard error of the mean. Interaction effects analysis was used to analyze differences between groups (*P* < 0.05 was considered statistically significant using one-way ANOVA). * represents *P* < 0.05；** represents *P* < 0.01；*** represents *P* < 0.001；**** represents *P* < 0.0001; NS, not significant.

In view of the serum hormone changes of mice in each group, we detected the key transcripts of glucose metabolism in muscle and liver. Consistent with the results of impaired glucose tolerance in intestinal FFAR4-deficient mice at the moment of ZT12, compared to Villin Cre control mice, liver *Glut2* and *Hk2* mRNA levels were significantly downregulated in Gko control mice (Fig. 6a and c); muscle *Glut1*, *Glut2*, *Pkm*, *Gyg*, and *Gsk3a* mRNA levels were significantly downregulated in Gko control mice (Fig. 6d and h). These results suggest that intestinal FFAR4 deficiency leads to a downregulation of liver glucose uptake and glycolytic capacity and a downregulation of muscle glucose uptake, glycolysis, and muscle glycogen synthesis capacity at ZT12. After the administration of *A. muciniphila* solution, liver *Pkm* mRNA levels and muscle *Glut2*, *Gyg*, and *Gsk3a* mRNA levels were significantly upregulated in Villin Cre mice (Fig. 6b, e, g and h); liver *Glut2*, *Pkm* mRNA levels, and muscle *Glut1*, *Glut2*, *Pkm*, *Gyg*, and *Gsk3a* mRNA levels were significantly upregulated in Gko mice (Fig. 6a, b, d and h).

## DISCUSSION

During the transition from subjective rest to subjective activity, the baseline glucose levels, insulin sensitivity, and glucose tolerance peak gradually in mice, preventing hypoglycemia during sleep and providing energy for activity after waking (3, 8). It has been noted that *Ffar3* and *Ffar2* mRNA expressions showed a diurnal rhythm in the colonic muscle layer (38). Our data verified the rhythmic expression of FFAR4 from *in vivo*. Like the diurnal pattern of glucose tolerance in mice, we found that colonic FFAR4 expression was upregulated as mice entered the active phase (Fig. S2). Moreover, both total *Ffar4* knockout and gut *Ffar4*-specific knockout mice severely affected the process of glucose utilization in the waking phase (ZT12). Impaired glucose tolerance at the daily light to dark transition (ZT12) in mice was similar to DP of diabetes in humans (9). Our data are the first to correlate FFAR4 with DP and may lead to a new understanding of the mechanisms of DP occurrence.

Total *Ffar4* knockout showed difference in growth hormone and glucagon-like peptide 1 levels at certain ZTs compared to control mice. Compared to control mice, gut-specific knockout mice had severely downregulated serum GLP-1 levels and showed no significant differences in growth hormone, which may be related to the function of FFAR4 in other organs or tissues. It is worth mentioning that FFAR4-specific agonists have been reported to upregulate serum GLP-1 levels in mice, which in turn increases insulin levels (39, 40). However, our data show that the decrease in GLP-1 levels caused by FFAR4 deficiency did not affect the change in serum insulin levels.

Free fatty acid receptor 4 (also known as GPR120) is a specific receptor for n3-polyunsaturated fatty acids (PUFAs). It has been reported that there exist several *FFAR4* mutations in diabetic populations (41 – 44); therefore, LCFAs acting as endogenous ligands may influence the risk of DP differently in these populations. Furthermore, we previously found that FFAR4 is not required for n3-PUFA-induced cell growth inhibition and apoptosis in cancer cells (45). FFAR4 may not participate in the regulation of glucose tolerance through LCFA, but may rather directly modulate the dysfunction of glucose tolerance at the daily light to dark transition (ZT12).

This study further suggested that the aberrant glucose tolerance induced by FFAR4 deletion was mediated by *A. muciniphila*. *Ffar4*-specific ligands, n3-PUFAs, are influential components of the diet and have been shown to significantly increase *A. muciniphila* abundance in the gut (46, 47). However, it is worth exploring which key *A. muciniphila*-derived metabolites affect the process of glucose metabolism. Previous studies have shown that *A. muciniphila* is an important source of SCFAs (48, 49), and we found that acetic acid abundance at ZT12 was disrupted in total *Ffar4* KO mice (*P* < 0.001) but not in gut *Ffar4*-specific knockout mice compared to control mice. Additionally, several studies have shown that *A. muciniphila* can significantly stimulate an increase in serum GLP-1 levels through secretory protein P9, an effective mediator of GLP-1 secretion (50 – 52). Similarly, we found that treatment with *A. muciniphila* immediately increased GLP-1 and insulin secretion and decreased glucagon and GH secretion in gut-specific knockout of *Ffar4* mice. Elevated serum GLP-1 levels in mice have been widely reported to be associated with increased insulin levels, insulin sensitization, and decreased glucagon levels. Serum growth hormone levels have also been reported to be associated with insulin sensitization.

**FIG 7:**
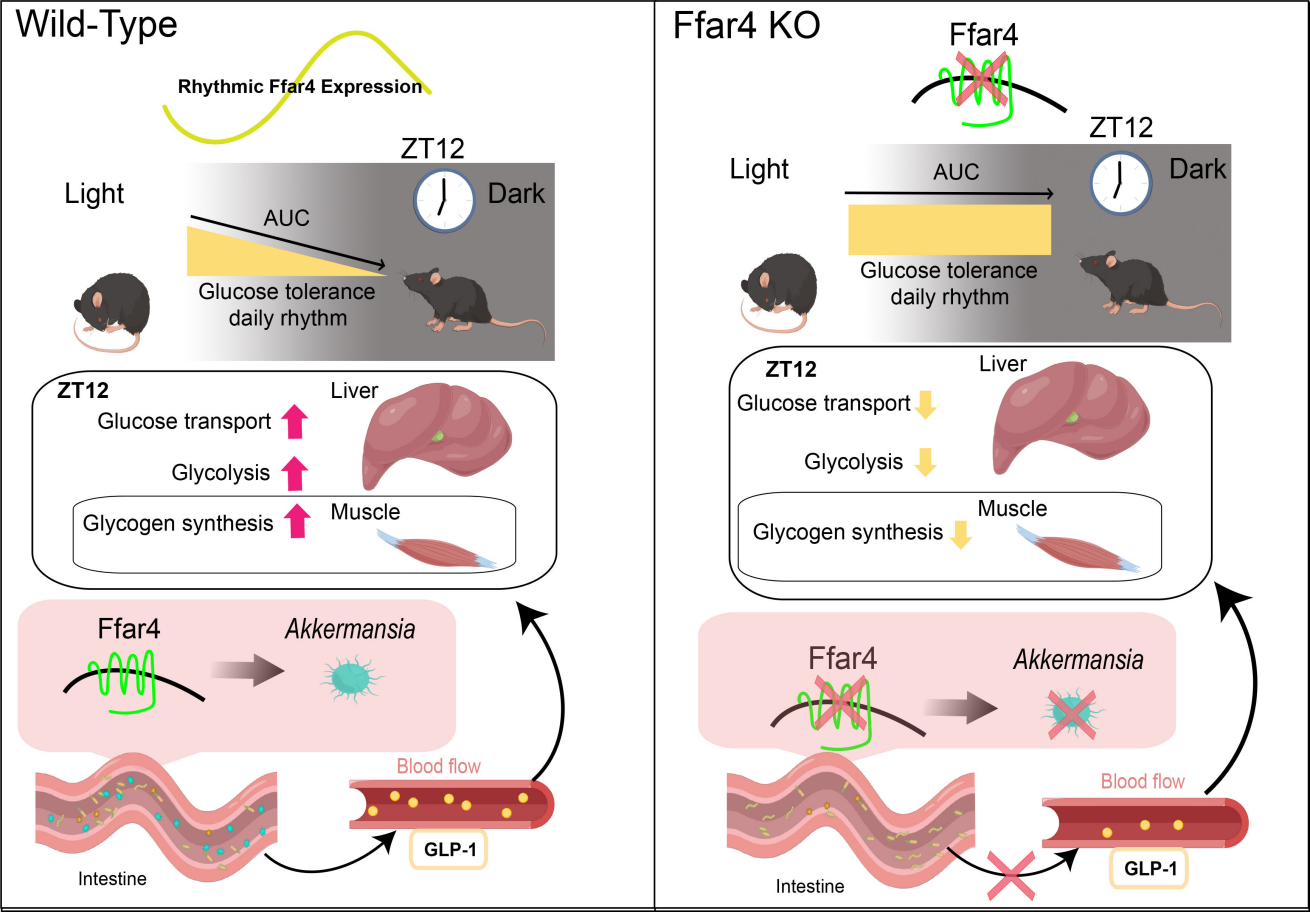
Conceptual summary. Intestinal *Ffar4* affects hormone secretion such as GLP-1 secretion via *Akkermansia muciniphila*, which can modify glucose metabolism in the liver and muscle of mice, affecting glucose tolerance at the daily light to dark transition. GLP-1, glucagon-like peptide 1.

Consistent with our hypothesis, intestinal FFAR4 deficiency, although not leading to the disappearance of the glucose tolerance rhythm in mice, caused a delay in the peak and trough of the glucose tolerance rhythm in mice, which was mainly caused by the impaired glucose tolerance in mice at ZT12 due to intestinal FFAR4 deficiency. Intestinal FFAR4 deficiency resulted in a downregulation of *A. muciniphila* abundance in the intestine at ZT12, and after pasteurized *A. muciniphila* supplementation, Gko mice showed improved glucose tolerance at ZT12, accompanied by changes in serum hormones and an increase in peripheral tissue glucose metabolism. In particular, the restoration of serum GLP-1 hormone in Gko mice after *A. muciniphila* supplementation increased serum insulin levels and improved liver and muscle glucose utilization. GLP-1 receptor is absent in muscle and liver tissue, so changes in glucose utilization in liver and muscle tissue should be mediated by GLP-1-affected insulin. At the same time, a decrease in growth hormone means that the resistance to insulin of mice is weakened, which also facilitates blood glucose utilization in mice. The decrease of serum glucagon level is beneficial to the gluconeogenesis process, which was also demonstrated by upregulation of gluconeogenic transcripts in muscle tissue of *A. muciniphila* supplemented Gko mice (Fig. 6; Fig. S6). The above results suggest that restoring the FFAR4-*A. muciniphila* pathway plays a key role in maintaining glucose tolerance levels at ZT12 and even glucose tolerance rhythm in mice (Fig. 7). Moreover, it is necessary to develop new strategies to modulate the activity of the FFAR4-*A. muciniphila* pathway *in vivo* as it may serve as a novel intervention for the management of glucose tolerance rhythm in the future.

## MATERIALS AND METHODS

### Animals

*Ffar4* total KO (RRID: MGI:7256540) mice were provided by Shanghai Bioray Lab. Floxed *Ffar4* (fl/fl, RRID: MGI: 7256541) and Villin-Cre mice (RRID: IMSR_JAX: 004586) mouse models were established by Shanghai Biomodel Organism. Tail tips of mice were digested with 100 µL of 50-mM NaOH at 95°C for 20 min. Next, 10 µL of Tris-HCl (pH 8.0) was added to neutralize the mixture. KO mice were genotyped by PCR using tail DNA with the forward primer CCCGGCATGTCCCCTGAGTGT and reverse primer TGGTCGCCCTTGACATCCGAGA at 95°C for 30 s and 63°C for 45 s for 40 cycles, generating an 87-bp fragment for the wild type and a 110-bp fragment for KO mice. *Ffar4* (fl/fl) mice were genotyped using the forward primer TGCTCTTTCTGGAGCTGTGT and the reverse primer AGAGATCAGAATGGACAACT at 95°C for 30 s, 58°C for 45 s, and 72°C for 30 s for 40 cycles, generating a 272-bp fragment for fl/fl and a 243-bp fragment for the wild type. Villin-Cre mice were genotyped using the forward primer AGCGATGGATTTCCGTCTCTGG and the reverse primer AGCTTGCATGATCTCCGGTATTGAA at 95°C for 30 s, 58°C for 45 s, and 72°C for 30 s for 40 cycles, generating a 272-bp fragment. Gut-specific *Ffar4* knockout mice (Gko) were generated by mating fl/fl mice with Villin-Cre mice. All mice were on a C57BL/6J background. Animals were housed and bred in a sterile-pathogen-free environment, subjected to a 12-hour light/12-hour dark cycle (07:00 lights on, 19:00 lights off), and provided access to water and food *ad libitum*.

### Animal experiments

Adult male mice were used in all experiments. These mice were litter mates or from litters born during the same time period. To test the impact of *A. muciniphila* on glucose tolerance in Gko mice, the mice were treated with pasteurized *A. muciniphila* [5 × 10^10^ colony forming units (CFU)] or the control medium for 6 weeks until the GTT was performed. Mice were euthanised and then tissue sampled after the GTT.

### Glucose tolerance test

Mice were fasted for 6 hours before the GTT. Tail blood glucose levels were measured immediately using a glucometer (OneTouch Ultra2), and a glucose injection corresponding to 0.8 g/kg of body weight was administered immediately afterward. Blood glucose levels were measured using glucometers at 15, 30, 60, 90, and 120 min. The tests at ZT12–14, ZT15–17, ZT18–20, and ZT21–23 were performed under dim red light.

### Serum hormone assay

After at least 1 week of recovery after GTT, mice blood was collected through orbital puncture in EDTA tubes in non-fasting conditions. Serum insulin, glucagon, corticosterone, and glucagon-like peptide 1 (GLP-1) total levels were measured using an ultra-sensitive mouse insulin enzyme-linked immunosorbent assay (ELISA) kit (90080, Crystal Chem), glucagon ELISA kit (10–1281-01, Mercodia), corticosterone ELISA kit (ADI-900–097, Enzo Life Sciences), mouse GLP-1 ELISA kit (81508, Crystal Chem), and growth hormone ELISA kit (EZRMGH-45K, Sigma), respectively.

### Quantitative real-time PCR

According to the manufacturer’s protocol, total RNA was isolated with RNAiso Plus (Takara). RNA was reverse-transcribed using Prime Script RT Master Mix (Takara). mRNA quantification was performed using a CFX Connect Real-Time PCR Detection System (Bio-Rad) and Ultra SYBR Mixture (CWBIO). qPCR included 40 cycles of denaturation at 95°C for 15 s, and annealing and extension at 60°C for 30 s. Animal experiment results were normalized to the housekeeping gene β-actin and were presented as 2^−ΔΔCt^. Genomic DNA of mice fecal samples collected at different times was extracted using the Fast DNA Spin kit for soil (MP, USA) following the manufacturer’s instructions. An amount of 20  ng of DNA was then used in qPCR reactions for *A. muciniphila* with specific primers, and for total bacteria for normalisation. The nucleotide sequences of primers used in this experiment are listed in Table 1.

**TABLE 1:** Primers used in qRT-PCR

Primer	Sequence
Mus β-actin forward primer	AGGAGTACGATGAGTCCGGC
Mus β-actin reverse primer	AGGGTGTAAAACGCAGCTCAG
Mus Ffar4 forward primer	AGTCAATCGCACCCACTTCC
Mus Ffar4 reverse primer	CCCAGCAGTGAGACGACAAA
Mus Glut1 forward primer	TCTGGACAAACCCACTTGTAA
Mus Glut1 reverse primer	CCAGCCATTTATATCTGCTTAGGT
Mus Glut2 forward primer	GGTGATGGGTCTCATTGGTGA
Mus Glut2 reverse primer	ACACGGGAGGCAAACAACTA
Mus Pkm forward primer	AGTGCCTGTACCTTGATGGC
Mus Pkm reverse primer	TACAAGCGTTGCTGGCCTAA
Mus Gyg1 forward primer	GAACCTGTGGTGGGACACTT
Mus Gyg1 reverse primer	CCTGAGACATGTTCCATCATTAGA
Mus Gsk3a forward primer	CGATGAACTGCGGAGACTCG
Mus Gsk3a reverse primer	GGATGGACAGTTCACCAGGAC
Mus Hk2 forward primer	ACCCGGGATGTTAGGCAAAG
Mus Hk2 reverse primer	ACCATTCTGAAACGCCGACT
*A. muciniphila* forward primer	CAGCACGTGAAGGTGGGGAC
*A. muciniphila* reverse primer	CCTTGCGGTTGGCTTCAGAT
UniF340 forward primer	ACTCCTACGGGAGGCAGCAGT
UniR514 reverse primer	ATTACCGCGGCTGCTGGC

### Western blotting

Distal colons of mice were lysed in RIPA buffer with 1 × protease inhibitor cocktail. Protein samples were separated using sodium dodecyl sulfate–polyacrylamide gel electrophoresis and electrically transferred to polyvinylidene fluoride membranes. Tris buffered saline with Tween (TBST) containing 5% skim milk was used to block the membranes for 2 hours. The membranes were washed four times with TBST and incubated overnight at 4°C with primary antibodies, including antiβ-ACTIN (Abcam, UK) and anti-FFAR4 (Sigma-Aldrich, USA). The next day, membranes were washed four times with TBST and incubated with horseradish peroxidase-conjugated secondary immunoglobulin G antibody for 2 hours at 25°C. For protein detection, the membranes were washed three times with TBST before imaging.

### 16S rRNA gene sequencing and analysis

Genomic DNA of mice fecal samples collected at different times (ZT0, ZT6, ZT12, and ZT18) was extracted using the Fast DNA Spin kit for soil (MP, USA) following the manufacturer’s instructions. Subsequently, the V3–V4 region (forward primer, 5-CCTACGGGNGGCWGCAG-3; reverse primer, 5-GGACTACHVGGGTATCTA AT-3) of the 16S rDNA gene fragments was amplified. The 50-µL reaction system contained 25 µL of premix Taq lus dye (TaKaRa, China), 0.5 µL of the forward primer, 0.5 µL of the reverse primer, 2 µL of the DNA template, and 22 µL of ddH_2_O. The amplification conditions were as follows: 95°C for 5 min, 40 cycles of 95°C for 30 s, 52°C for 30 s, 72°C for 30 s, and maintaining at 12°C for 10 min. The PCR products were purified after amplification using the QIAquick PCR Purification kit (Qiagen, USA). An Agilent 2100 Bioanalyzer (Agilent Technologies, Palo Alto, CA, USA) and Qubit (version 2.0; Applied Biosystems, Carlsbad, CA, USA) were used to verify and quantify the DNA libraries; moreover, paired-end sequencing was performed using the Illumina Miseq platform. After sequencing, Quantitative Insights into Microbial Ecology 2 (version 2019.7) was used to analyze the high-quality sequences (53). A naive Bayes classifier was trained using the SILVA reference database (version 132) (54). DADA2 was used for quality control, to remove low quality sequences, chimeras, and generate OTU tables with 100% similarity (now called feature tables)(55). Phylogenetic trees were constructed *de novo*; the construction process includes multiple sequence alignment, removal of high variation sites, construction of an unrooted phylogenetic tree, and transformation of the unrooted phylogenetic tree into a rooted phylogenetic tree. Diversity analyses were performed after samples were rarefied to a common sampling depth of 5623. Differential microbial analysis was performed using QMD (a method for quantifying differences in absolute microbial abundance) (56).

### Culture and pasteurization of *A. muciniphila*

*A. muciniphila* (ATCC BAA-835) was cultured in brain-heart infusion broth containing 50-mg/L L-cysteine and 500-mg/L mucoprotein under strict anaerobic conditions. A representative culture stock was used to determine the CFU per milliliter under anaerobic conditions by plate counting using mucin media containing 1% agarose. This culture was diluted with anaerobic PBS containing 2.5% glycerol to a final concentration of 2.5 × 10^10^  CFU/mL. Then, *A. muciniphila* was inactivated by pasteurization for 30  min at 70°C. After pasteurization, no viable *A. muciniphila* could be recovered in the culture.

### Gas chromatography-mass spectroscopy

Fresh feces of mice were collected at ZT12, and 500 µL of saturated sodium chloride solution was added immediately. After shaking treatment, 10% sulfuric acid solution was added for acidification, and the SCFAs were extracted using anhydrous ether. The ether phase was separated and anhydrous sodium sulfate was added to absorb the residual moisture. The ether phase was analyzed using gas chromatography-mass spectroscopy (TRACE1310-TSQ 8000 Evo; Thermo Fisher Scientific, USA). An Rtx-5 MS capillary tube chromatographic column (0.25 mm ×30 m, 0.25 µm) was purchased from Restek, USA, and water for experiments was produced using a Milli-Q8 ultrapure water generator (Millipore Co., USA). Concentrated sulfuric acid, acetic acid, propionic acid, and n-butyric acid solutions of analytically pure grade were purchased from China National Pharmaceutical Group Co., Ltd. (Sinopharm).

### Statistical analysis

Cosine analysis was used to analyze the circadian rhythm of the data; we estimated the loss or gain of rhythms and circadian properties including amplitude, phase, and mesor. *P* < 0.05 was considered statistically significant. Animal data were analyzed using two-way analysis of variance (ANOVA) and one-way ANOVA using SPSS (SPSS Inc.). For sequencing data, the R platform (http://www.r-project.org/) was used for statistical analysis. Differences in the diversity of intestinal flora were compared using the function of the Kruskal–Wallis test of the vegan package of R. All plots are presented as the mean ± standard error of the mean. *P* < 0.05 was considered statistically significant.

## Data Availability

The data that support the findings of this study are available from the corresponding author upon reasonable request. The 16S rRNA gene sequences are available in the NCBI SRA database under accession no. PRJNA1001966.

## SUPPLEMENTAL MATERIAL

Figure S1 msystems.00573-23-s0001.tif Identification of mouse tail genotypes.

Figure S2 msystems.00573-23-s0002.tif
*Ffar4* mRNA and *FFAR4* protein expression oscillations showed a circadian rhythm in the colons of mice.

Figure S3 msystems.00573-23-s0003.tif Identification of mouse tail genotypes.

Figure S4 msystems.00573-23-s0004.tif Comparison of SCFA levels in mouse intestinal feces.

Figure S5 msystems.00573-23-s0005.tif Relative abundance of *A. muciniphila*.

Figure S6 msystems.00573-23-s0006.tif Serum insulin, glucagon, GC, GH, and GLP-1 levels after gavage with pasteurized *A. muciniphila*.

Legends msystems.00573-23-s0007.doc Supplemental figure legends.

ASM does not own the copyrights to Supplemental Material that may be linked to, or accessed through, an article. The authors have granted ASM a non-exclusive, world-wide license to publish the Supplemental Material files. Please contact the corresponding author directly for reuse.
