# Supplementary figures and images for: Akkermansia muciniphila supplementation improves glucose tolerance in intestinal Ffar4 knockout mice during the daily light to dark transition
Source: mSystems. 2023 Oct 3;8(5):e00573-23. doi: 10.1128/msystems.00573-23 (PMC10654094; doi:10.1128/msystems.00573-23)

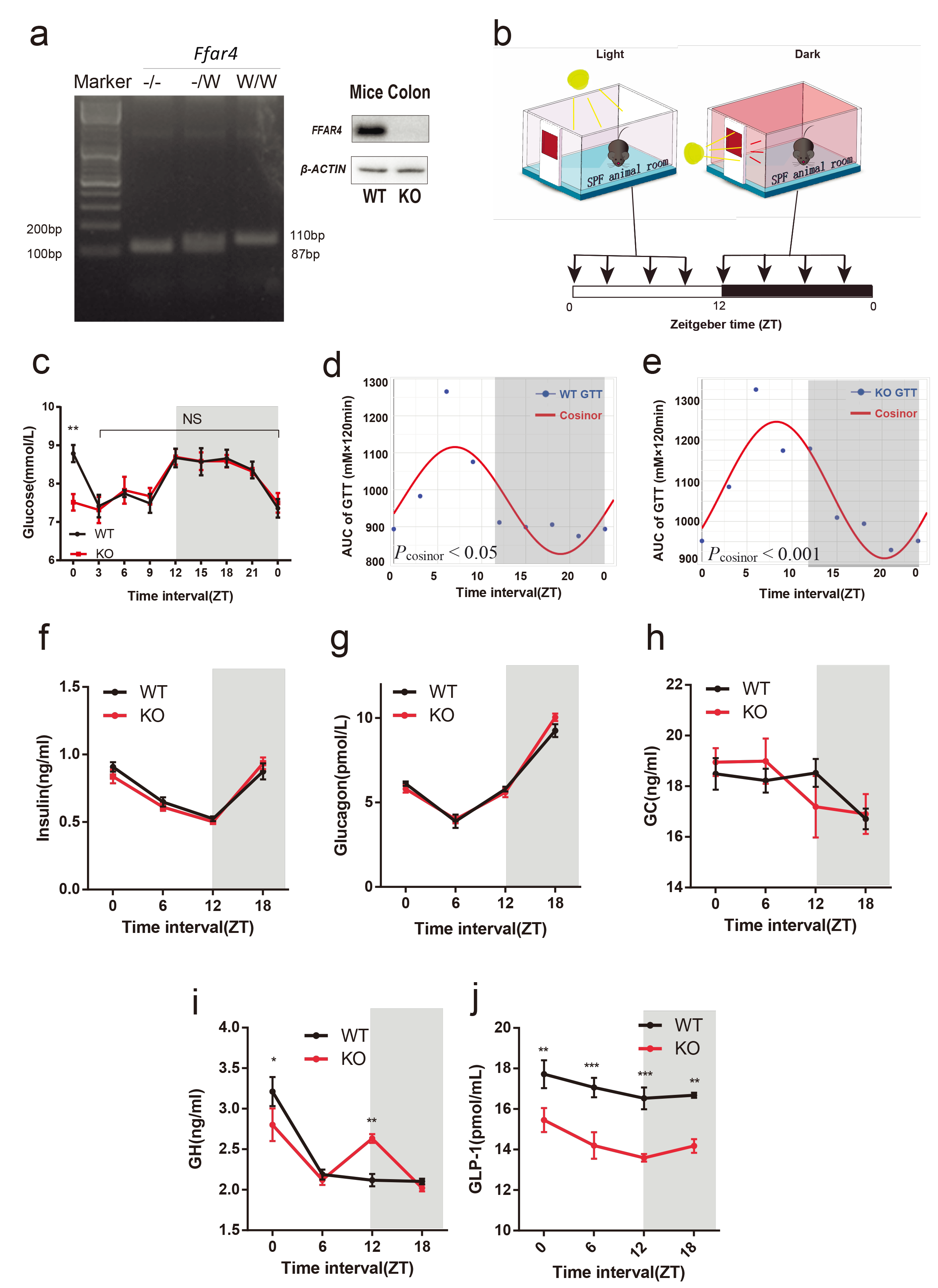

Supplement: Figure S1 — Identification of mouse tail genotypes. [file msystems.00573-23-s0001.tif]

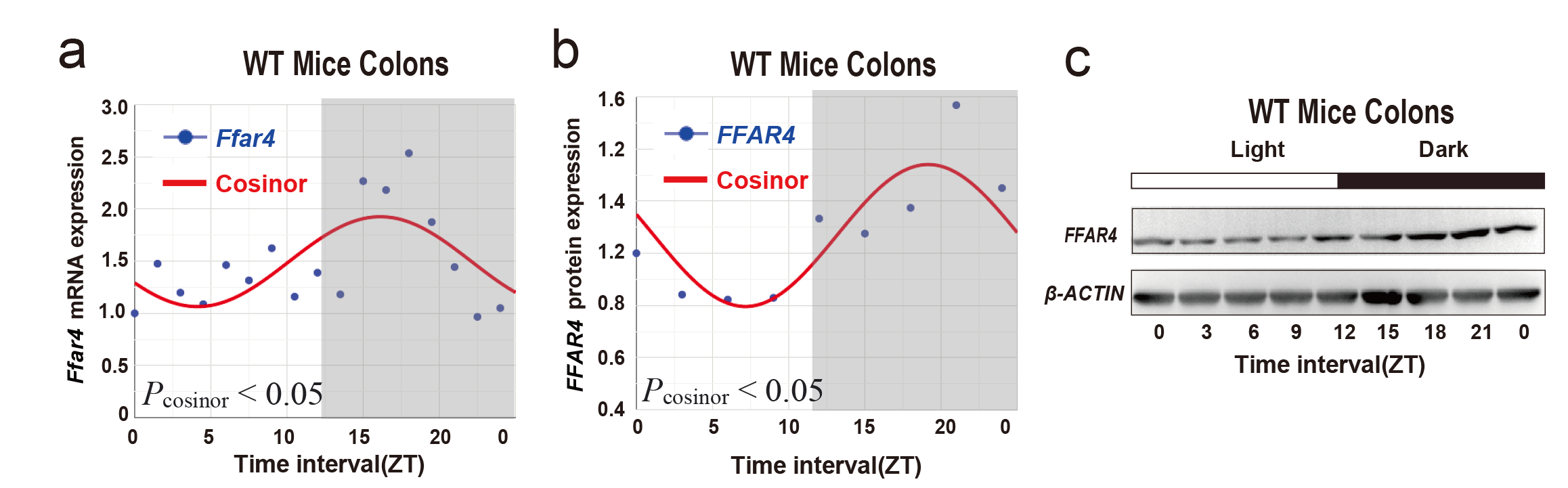

Supplement: Figure S2 — Ffar4 mRNA and FFAR4 protein expression oscillations showed a circadian rhythm in the colons of mice. [file msystems.00573-23-s0002.tif]

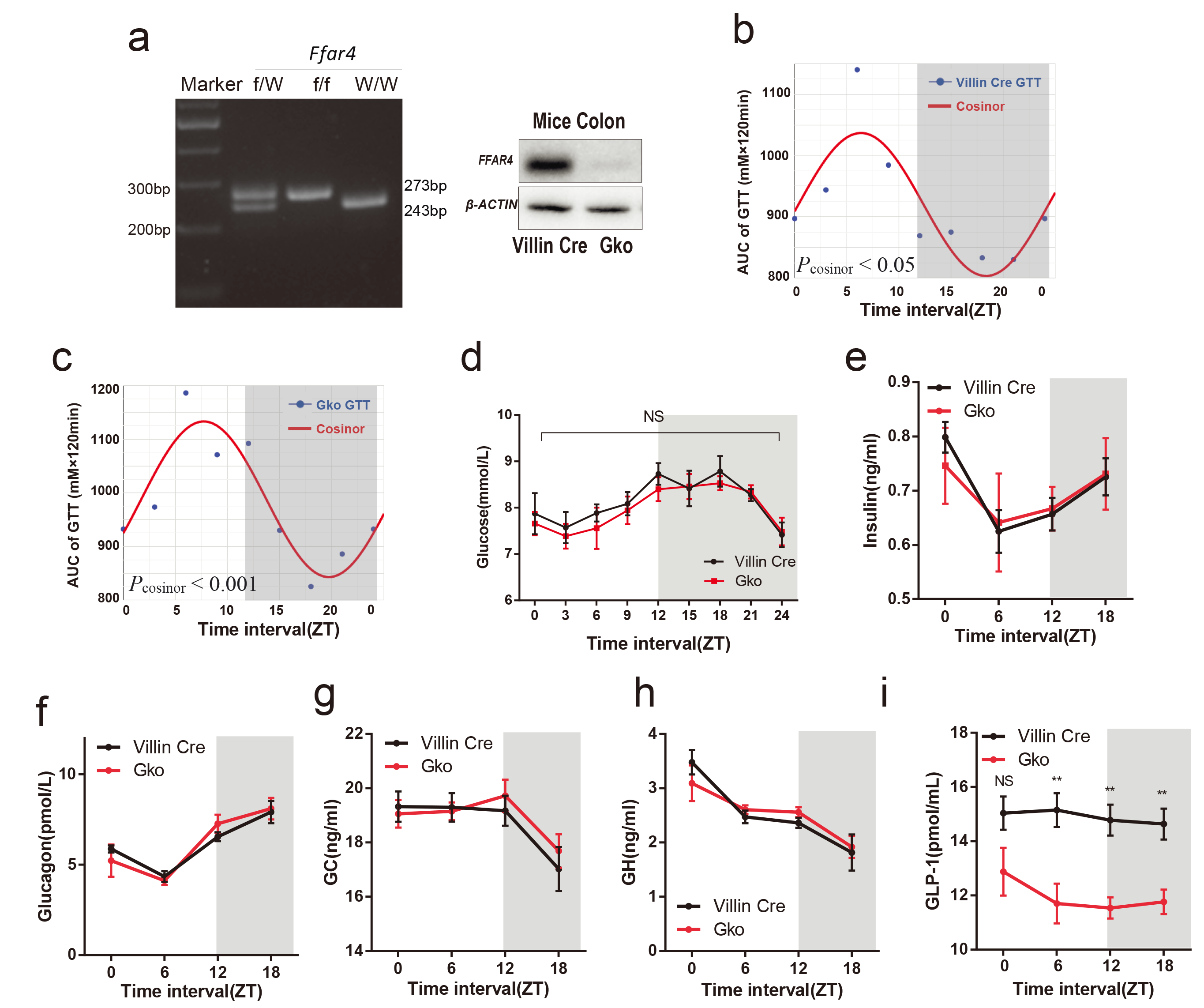

Supplement: Figure S3 — Identification of mouse tail genotypes. [file msystems.00573-23-s0003.tif]

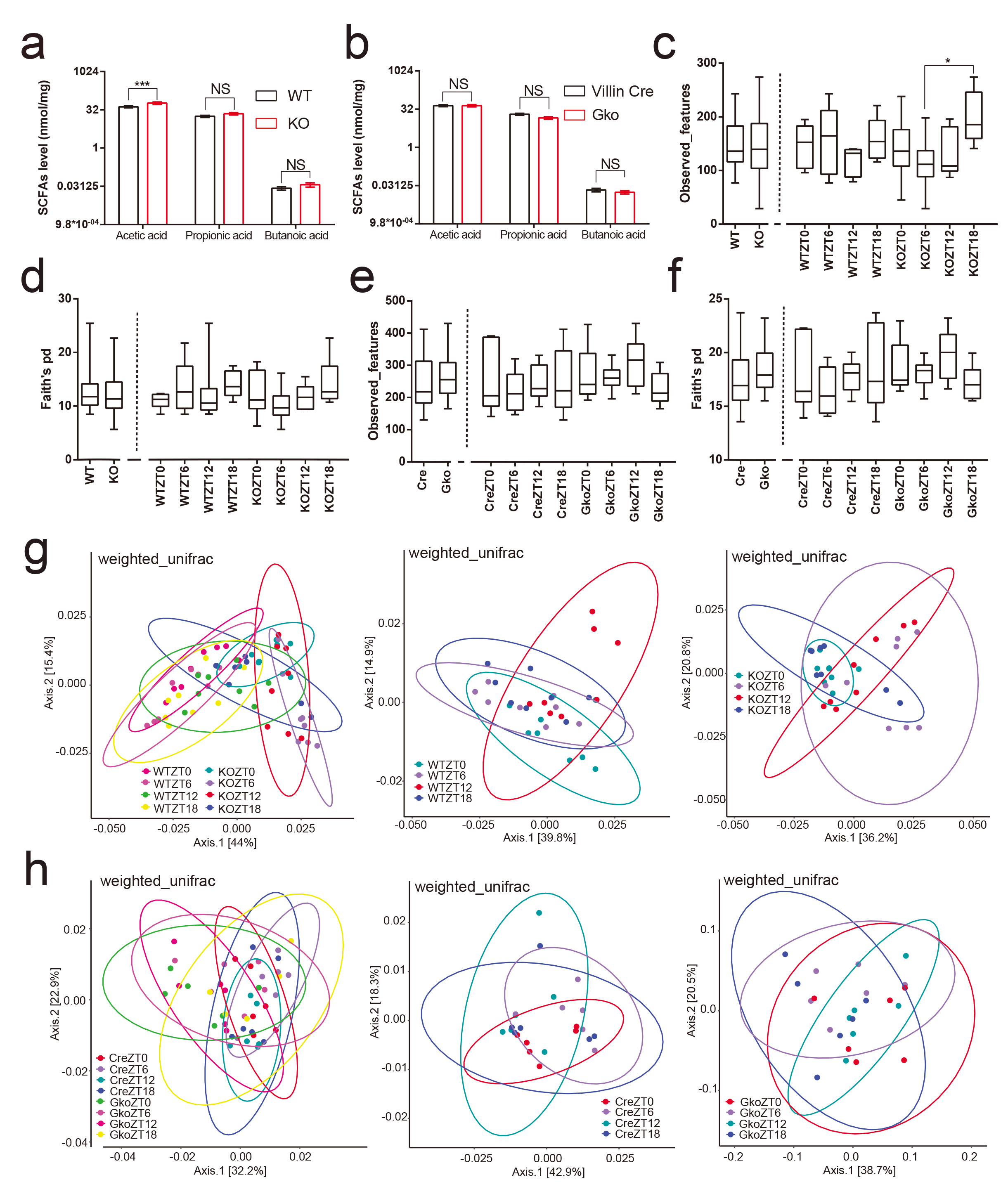

Supplement: Figure S4 — Comparison of SCFA levels in mouse intestinal feces. [file msystems.00573-23-s0004.tif]

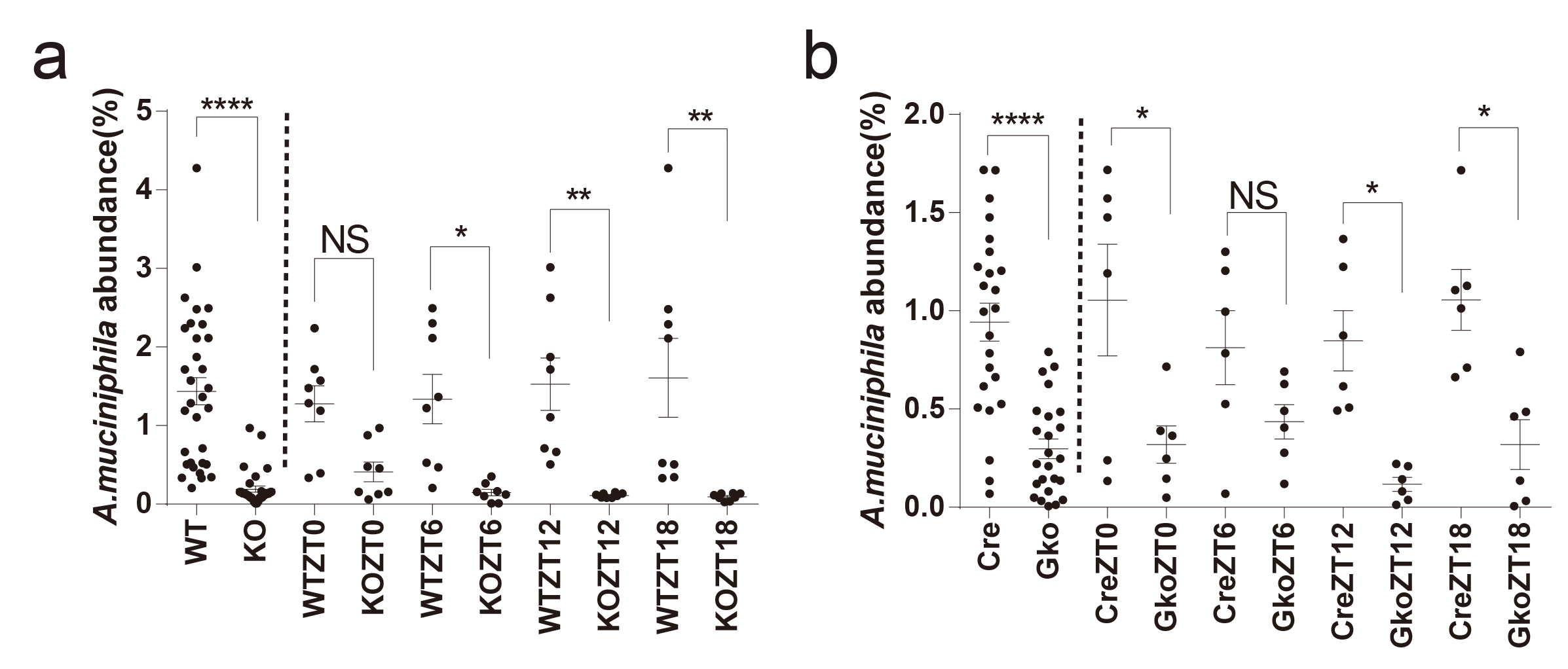

Supplement: Figure S5 — Relative abundance of A. muciniphila. [file msystems.00573-23-s0005.tif]

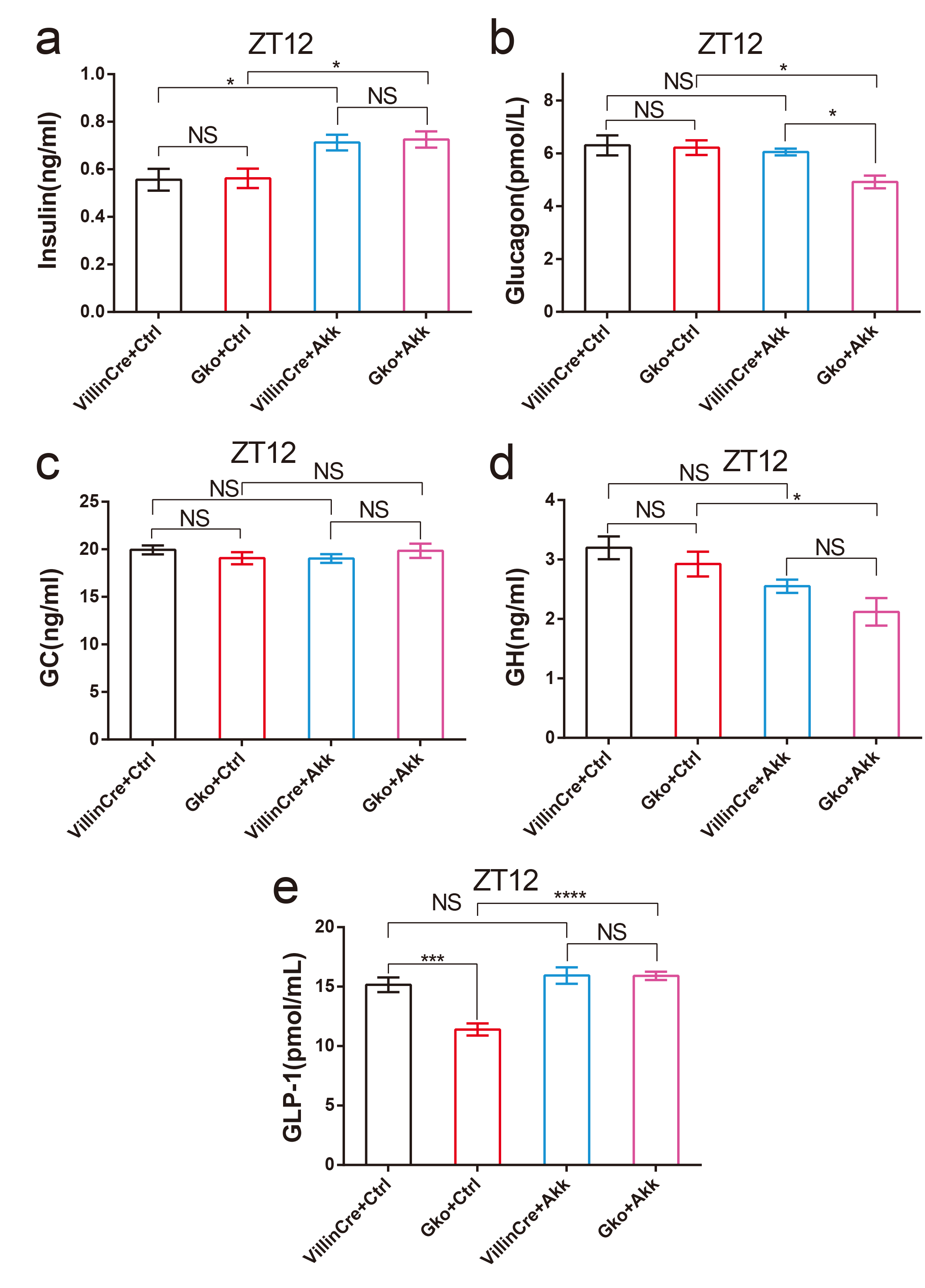

Supplement: Figure S6 — Serum insulin, glucagon, GC, GH, and GLP-1 levels after gavage with pasteurized A. muciniphila. [file msystems.00573-23-s0006.tif]
