# Supplementary material for: Akkermansia muciniphila supplementation improves glucose tolerance in intestinal Ffar4 knockout mice during the daily light to dark transition
Source: mSystems. 2023 Oct 3;8(5):e00573-23. doi: 10.1128/msystems.00573-23 (PMC10654094; doi:10.1128/msystems.00573-23)
Supplement: Legends — Supplemental figure legends. [file msystems.00573-23-s0007.doc]

**Supplemental Data**

**Supplementary Figure 1:** Identification of mice tail genotypes in wild-type (WT), heterozygous, total *Ffar4* knockout (KO) mice by agarose electrophoresis and detection of FFAR4 protein expression in WT

&KO mice colons **(a).** Glucose tolerance test (GTT) experimental was designed at different time points (ZT0–2; ZT3–5; ZT6–8; ZT9–11; ZT12–14; ZT15–17; ZT18–20; ZT21–23); GTTs at ZT12–14, ZT15–17, ZT18–20, and ZT21–23 were conducted in a dimly lit environment **(b).** 24-h blood glucose test (WT/KO mice, n=7) between WT and KO mice were recorded **(c).** The blood glucose areas under the curve (AUCs) of mice after glucose injection were shown in **(d-e)** using a cosine analysis (P < 0.05 for WT mice, P < 0.01 for WT/KO mice, n=13 per ZT). Serum insulin, glucagon and glucocorticoid (GC), growth hormone (GH), glucagon-like peptide 1 (GLP-1) levels were shown in **(f-j)** (P < 0.05 using two-way ANVOA; NS, not significant, WT/KO mice, n=6 per ZT). Data are expressed as the mean ± standard error of the mean.

**Supplementary Figure 2:** *Ffar4* mRNA and *FFAR4* protein expression oscillations showed a circadian rhythm in the colons of mice **(a-c)** (both P < 0.05 using a cosine analysis, wild-type [WT] mice, n=51, n=3 mice per ZT).

**Supplementary Figure 3:** Identification of mice tail genotypes in Villin-Cre, heterozygous, gut-specific *Ffar4* knockout (Gko) mice by agarose electrophoresis and detection of FFAR4 protein expression in Villin Cre and GKo mice colons **(a).** The blood glucose areas under the curve (AUCs) of mice after glucose injection were shown in **(d-e)** using a cosine analysis (P < 0.05 for Villin-Cre mice, P < 0.01 for Gko mice, Villin-Cre / Gko mice, n=8 per ZT). Serum insulin, glucagon and glucocorticoid (GC), growth hormone (GH), glucagon-like peptide 1 (GLP-1) levels were shown in **(i-m)** (P < 0.05 using two-way ANVOA; NS, not significant, Villin-Cre/Gko mice, n=6 per ZT). Data are expressed as the mean ± standard error of the mean.

**Supplementary Figure 4:** Comparison of short-chain fatty acid (SCFA) levels of WT, Villin-Cre, KO, Gko mice intestinal feces at ZT12 was shown in **(a, b)** (WT/KO/Villin-Cre/Gko mice, n=7). Data are expressed as the mean ± standard error of the mean. Interaction effects analysis was used to analyze differences between groups (P < 0.05 using one-way ANVOA; NS, not significant). The alpha diversity (observed features & faith’s pd) of WT, Villin-Cre, KO, Gko mice were shown in **(c-f)**. Beta diversity analysis (weighted unifrac) between different ZT were shown in **(g-h)**. (Wild-type [WT]/knockout [KO] mice, n=8 per ZT; Villin-Cre/Gko mice, n=6 per ZT).

**Supplementary Figure 5:** Percent relative abundance of A. muciniphila in regard to total bacteria as determined by qPCR (Wild-type [WT]/knockout [KO] mice, n=8 per ZT; Villin-Cre/Gko mice, n=6 per ZT) **(a, b)**. Data are expressed as the mean ± standard error of the mean.

**Supplementary Figure 6:** Serum insulin, glucagon, glucocorticoid (GC), growth hormone (GH) and glucagon-like peptide 1 (GLP-1) levels after gavage with pasteurized *Akkermansia muciniphila* between four groups were shown in **(a-e)** (Villin Cre+Ctrl/Villin Cre+Akk/Gko+Ctrl/Gko+Akk, n=6 per ZT, P < 0.05 using two-way ANVOA; NS, not significant).
